# On the Use of Solid Lipid Nanoparticles for Delivering Lavender Hydroalcoholic Extract as an Antioxidant to NMRI Mice Spermatozoa During Handling, Cryopreservation, and Thawing

**DOI:** 10.1002/rmb2.12699

**Published:** 2025-11-19

**Authors:** Zahra Asadi, Faranak Aghaz, Saba Jalilian, Saeed Khazayel, Somayeh Rahimi, Zohreh Rahimi, Elham Arkan, Asad Vaisi‐Raygani

**Affiliations:** ^1^ Department of Clinical Biochemistry, Medical School Kermanshah University of Medical Sciences Kermanshah Iran; ^2^ Students Research Committee Kermanshah University of Medical Sciences Kermanshah Iran; ^3^ Nano Drug Delivery Research Center, Health Technology Institute Kermanshah University of Medical Sciences Kermanshah Iran; ^4^ Department of Research and Technology Kermanshah University of Medical Sciences Kermanshah Iran; ^5^ Fertility and Infertility Research Center, Health Technology Institute Kermanshah University of Medical Sciences Kermanshah Iran

**Keywords:** cryopreservation, lavender hydroalcoholic extract, oxidative stress, solid lipid nanoparticles, sperm quality

## Abstract

**Purpose:**

This study investigated the cryoprotective and antioxidant effects of lavender hydroalcoholic extract‐loaded solid lipid nanoparticle (LHE‐SLN) during handling, freezing, and thawing of NMRI mouse sperm.

**Methods:**

LHE‐SLNs were synthesized using the self‐assembly method. After evaluating their physicochemical characteristics, NMRI mouse sperm were exposed to four concentrations (1.5, 3, 4.5 and 10 μg/mL) of LHE‐SLN in handling, freezing and post‐thaw incubation. After each step, sperm viability, motility and DNA fragmentation were assessed. The activities of antioxidant enzymes, including superoxide dismutase (SOD), catalase (CAT), and glutathione peroxidase (GPx), as well as the levels of nitric oxide (NO), were measured. The gene expression of *Sod1*, *Sod2*, *Gpx*, *Cat*, *Bax*, *Bcl2*, and *Casp3* was analyzed using quantitative real‐time PCR (qRT‐PCR).

**Results:**

The nanoparticles were spherical, with an average size of 235.8 ± 11.06 nm and a zeta potential of −21.7 ± 5.35 mV. All experiments showed increased cell viability and motility, reduced DNA fragmentation, and elevated NO levels. The activity of SOD, CAT, and GPX was significantly enhanced. Additionally, antioxidant genes were upregulated, while pro‐apoptotic genes were downregulated.

**Conclusion:**

These findings suggest that LHE‐SLNs, particularly at 1.5 μg/mL, can effectively reduce oxidative stress, potentially enhancing sperm preservation outcomes through sustained delivery of antioxidants.

## Graphical abstract

Lavender hydroalcoholic extract‐loaded SLN could improve the quality of NMRI mouse sperm during handling, freezing, and thawing processes.

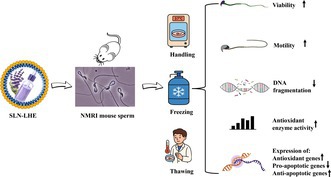

## 1 Introduction

Antioxidants are molecules that neutralize and scavenge reactive oxygen species (ROS), including hydroxyl radicals (OH﮲), superoxide anions (O_2_﮲^−^), and hydrogen peroxide (H_2_O_2_) [1]. These free radicals can damage all types of biomolecules, such as proteins, nucleic acids, fats, and starches. An excess of free radicals or a low concentration of antioxidants can disrupt the balance between antioxidants and prooxidants, leading to a condition known as oxidative stress (OS) [2].

Cryopreservation is a well‐known exogenous source of ROS. This process involves freezing cells, such as sperm, for long‐term storage [3]. Sperm are particularly vulnerable to OS caused by the freeze–thaw process. Additionally, even storing sperm at physiological temperatures (e.g., 37°C) can lead to oxidative damage [4]. These vulnerabilities arise from the limited capacity of sperm to repair themselves, their relatively weak antioxidant defense systems, and the high content of polyunsaturated fatty acids (PUFAs) in their membranes. OS negatively impacts sperm quality by compromising membrane integrity, viability, motility, DNA stability, and mitochondrial function, ultimately reducing their fertilization potential [5].

To overcome the detrimental effects of OS on spermatozoa, the use of antioxidants, particularly those derived from plants, has emerged as a promising strategy [6, 7]. *Lavandula angustifolia* is a medicinal herb belonging to the *Lamiaceae* (formerly *Labiatae*) family, with over 30 species naturally growing in the Middle East, southern Europe, Africa, and large areas of Asia [8]. This herb has traditionally been known for its antiseptic, antispasmodic, tranquilizing, pain‐relieving, and wound‐healing properties [9]. Additionally, lavender is recognized for its anti‐inflammatory, antimicrobial, antitumor, analgesic, and antioxidant activities [10].

Numerous studies have shown that the hydroalcoholic extract of lavender exhibits potent antioxidant activity through free radical scavenging, metal ion chelating, and prevention of lipid peroxidation [11, 12, 13, 14]. Hydroalcoholic extracts are generally rich in substances such as phenolic compounds and flavonoids, which are known for their strong antioxidant activity [15]. However, the use of these materials is limited due to their poor bioavailability and stability. Furthermore, to maximize therapeutic effectiveness, they need to be applied repeatedly and/or in high doses, which could be toxic to living systems and lead to adverse side effects [16].

Currently, there is a focus on using nanocarriers for the specific delivery of plant extracts as an alternative approach [17]. Solid lipid nanoparticles (SLNs) are particles with an average size of 50–1000 nm made using biodegradable, biocompatible, and non‐toxic lipids. They are being used as nanocarriers for the targeted delivery of plant extracts due to their safety and minimal organic solvent requirements for synthesis [18]. The solid matrix of SLNs allows for sustained drug release, protects the drug from degradation, and enhances its stability. Additionally, SLNs can increase drug solubility in water, membrane permeability, absorption, and pharmacological activity [19].

In the present research, we aimed to synthesize the lavender hydroalcoholic extract‐loaded SLN (LHE‐SLN) as an antioxidant supplement. We also investigated the effects of this nanosystem on the quality of NMRI mouse sperm after adding it to the media during handling, freezing, and thawing processes. In this regard, we investigated viability, motility, DNA fragmentation, nitric oxide (NO) production, and the activity of antioxidant enzymes, including superoxide dismutase (SOD), catalase (CAT), and glutathione peroxidase (GPx). Additionally, we evaluated the expression levels of antioxidant and apoptosis‐related genes, specifically *Gpx*, *Cat*, *Sod1*, *Sod2*, *Bcl2*, *Bax*, and *Casp3*.

## 2 Materials and Methods

### 2.1 Materials

All chemicals and reagents were purchased from Sigma‐Aldrich Chemical Co. (St. Louis, USA) unless otherwise stated. Milli‐Q (Millipore, Bedford, MA, USA) was used to generate Ultrapure water (UP‐water).

### 2.2 Plant Material and Extraction

The dried aerial parts of lavender were purchased from local markets and identified by a botanist from the Faculty of Pharmacy at Kermanshah University of Medical Sciences. Extraction was performed using the maceration method [20]. In brief, 10 g of dried lavender powder was macerated in 100 mL of ethanol 70% (v/v) with stirring (100 rpm at room temperature) for 24 h. Then the macerate was filtered through Whatman paper filters, and the solvent was evaporated through a rotary evaporator at 50°C and 150 rpm. The resulting extract was placed in the oven at 40°C for 12 h to complete the drying. The extract was collected and stored at 4°C until use.

### 2.3 SLN Preparation

The LHE‐SLNs were prepared by the ultrasonic self‐assembly method [21]. To obtain the aqueous phase, the surfactant Tween 80 (10 μL) was added to UP‐water (10 mL), and the mixture was magnetically stirred at 600–800 rpm at 50°C for 30 min. The lipid phase was also prepared by dissolving 10 mg of stearic acid in dichloromethane. Then, 3 mg of lavender hydroalcoholic extract (LHE) was dissolved in methanol (1 mL) and after filtration through a filter with a pore size of 0.22 μm, the solution was mixed with the lipid phase. Then, the final lipid phase was added dropwise to the aqueous phase under ultrasonic treatment (80 W) using a probe sonicator. The blank SLNs (B‐SLN) were synthesized using the same method, without the addition of any drug.

### 2.4 Characteristics of SLNs

The zeta potential, mean size, and polydispersity index (PDI) of SLNs were measured using dynamic light scattering (DLS) with a Zetasizer (Nano‐ZS, Malvern Co., United Kingdom). The average of three measurements was recorded. In addition, the structure and surface features of the SLNs were examined using a scanning electron microscope (SEM). To do this, freshly prepared SLNs were mixed with ultrapure water (1:10 ratio), and a few drops of the mixture were placed on a cover glass and left to dry. The dried SLNs were coated with gold–palladium membranes and then analyzed using an SEM (model SU3500, Hitachi Co., Japan).

### 2.5 The Evaluation of Drug Loading (DL) and Drug Entrapment Efficiency (EE)

To calculate EE (%) and DL (%), the LHE‐SLNs were ultracentrifuged at 30,000 *g* and 25°C for 20 min using an Optimal L‐90 k ultracentrifuge (Beckman Coulter Co., USA). The supernatant was then collected, and its absorbance was read at 221 nm with a UV spectrophotometer (Mini 1240, Shimadzu Co., Japan). The quantity of free Lavender hydroalcoholic extract was determined using a standard curve, and EE (%) and DL (%) were calculated using the following formulas [22]:

$$\mathrm{DL}\;\left(\%\right)=\frac{\text{Total amount of drug added}-\text{Free amount of drug}\;}{\text{Total weight of nanoparticles}}\times 100$$

$$\mathrm{EE}\;\left(\%\right)=\frac{\text{Total amount of drug added}-\text{Free amount of drug}}{\text{Total amount of drug added}}\times 100$$

### 2.6 Fourier‐Transformed Infrared (FT‐IR)

The freshly synthesized B‐SLN and LHE‐SLN were freeze‐dried to obtain a solid form. Subsequently, 5–6 mg of each sample, along with the LHE, were mixed with 100 mg of spectroscopy‐grade potassium bromide (Sigma Aldrich) and pressed into a disk [23]. The chemical composition of the samples was analyzed using an FT‐IR spectrophotometer (IR Prestige‐21, Shimadzu Co., Japan) over a wavenumber range of 4000–400 cm^−1^ at a resolution ratio of 4 cm^−1^.

### 2.7 In Vitro Drug Release

The dialysis bag diffusion technique was used to investigate the in vitro drug release [24]. Before the experiments, LHE‐SLNs were subjected to ultracentrifugation (Optimal L‐90 k, Beckman Coulter Co., USA) at 30,000 *g* for 20 min at 25°C. The supernatant was then discarded, and the LHE‐SLN pellet was resuspended in 4 mL of PBS. The resulting solution was placed in a dialysis bag with a molecular weight cut‐off of 12 kDa. Both ends of the bag were tied, and it was placed into a beaker containing 80 mL of PBS as the diffusion medium. The setup was maintained at 37°C for 48 h under stirring at 100 rpm to evaluate the membrane permeation kinetics. At predetermined time intervals (0, 3, 6, 16, 22, 25, 40, 45, and 48 h), 1 mL of the diffusion medium was withdrawn, and the same volume of fresh buffer was replaced. The collected samples were then analyzed at 221 nm using a UV spectrophotometer (Mini 1240, Shimadzu Co., Japan). The release percentage was calculated using the standard curve of Lavender.

### 2.8 Drug Release Kinetics

The cumulative amounts of LHE release from the SLN at different time intervals (0, 3, 6, 16, 22, 25, 40, 45 and 48 h) were fitted with the zero‐order kinetic model, first‐order kinetic model, Higuchi model and Korsmeyer‐Peppas model to determine the mechanism of LHE release [25].

### 2.9 Experimental Design

This study consists of three separate experiments involving the handling, freezing, and thawing of mouse sperm. Spermatozoa from NMRI mice were used for each experiment. Thirty adult male NMRI mice (6 to 8 weeks) were randomly divided into five groups, with six mice in each group (four treatment concentrations and one control group without any treatment). Each of the procedures was conducted in triplicate. All institutional and national guidelines for the care and use of laboratory animals were followed. The first animals were euthanized via cervical dislocation. The cauda epididymis and vasa deferentia were collected in Ham's F‐10 medium supplemented with 10% human serum albumin. Sperm were isolated using the swim‐up method after 1‐h incubation. The quality of the collected sperm was assessed according to World Health Organization (WHO) guidelines, focusing on motility, morphology, and viability using a light microscope [22]. Only high‐quality samples were used for the handling, freezing and thawing processes.

During handling, sperm samples were kept in microtubes containing a medium with four different concentrations of LHE‐SLN (1.5, 3, 4.5, and 10 μg/mL) for 1 h at 37°C and 5% CO_2_. After this period, the samples were centrifuged at 300 *g* for 7 min and evaluated for several parameters, including viability, motility, DNA fragmentation, NO production, and the activity of SOD, CAT, and GPx. Additionally, the expression levels of antioxidant and apoptosis‐related genes, specifically *Gpx*, *Cat*, *Sod1*, *Sod2*, *Bcl2*, *Bax*, and *Casp3*, were assessed using real‐time polymerase chain reaction (RT‐PCR).

During the second experiment, sperm were frozen in the presence of different concentrations of LHE‐SLN (1.5, 3, 4.5, and 10 μg/mL) in cryopreservation medium for 2 weeks. Initially, samples were treated with the specified concentrations of LHE‐SLN, and then the freezing medium (Ravan Sazeh Co., Tehran, Iran) was gradually added until a final ratio of 1:0.7 was achieved. The cryovials containing the samples were incubated at 37°C for 10 min. After this, they were exposed to liquid nitrogen vapor for 15 min before being stored in liquid nitrogen at −196°C. After 14 days, the samples were thawed, centrifuged at 300 *g* for 7 min, and several parameters were analyzed, including motility, viability, DNA fragmentation, and the activities of SOD, CAT, and GPx, as well as NO. production. Additionally, the expression of antioxidant and apoptosis‐related genes, including *Gpx*, *Cat*, *Sod1*, *Sod2*, *Bcl2*, *Bax*, and *Casp3*, was evaluated.

The last experiment involved freezing NMRI mouse sperm without any treatment for 2 weeks. After this freezing period, the cryovials were rapidly thawed in a container of tap water maintained at 35°C–37°C for 3–5 min until all ice had melted. The sperm suspension was then gradually treated with pre‐warmed (37°C) Ham's F10 medium supplemented with 10% human serum albumin, as the thawing media, containing different concentrations of LHE‐SLN (1.5, 3, 4.5, and 10 μg/mL). After 1 h of incubation at 37°C and 5% CO_2_, the samples were centrifuged at 300 *g* for 7 min and then assessed for motility, viability, DNA fragmentation, NO production, and the activities of the antioxidant enzymes SOD, CAT, and GPx. Moreover, the expression of antioxidant and apoptosis‐related genes, including *Bcl2*, *Bax*, *Casp3*, *Gpx*, *Cat*, *Sod1*, and *Sod2*, was assessed.

### 2.10 Sperm Motility and Viability Analyses

The sperm motility was analyzed according to the World Health Organization (WHO) guidelines [26]. Firstly, 10 μL of each sample was placed on a slide, and at least 200 sperm from each sample was assessed using optical microscopy (400×). The sperm were then classified into three categories: progressive, non‐progressive, and immotile. Progressive motility was defined as spermatozoa demonstrating active forward movement, either in a linear direction or in a broad circular path, regardless of speed. Spermatozoa exhibiting slow or irregular movement patterns were classified as non‐progressive.

To assess sperm viability, Eosin staining was used. Initially, 40 μL of each sperm suspension was mixed with 10 μL of a 0.5% Eosin solution. After 10 min, the samples were evaluated using an optical microscope (400×). Unstained cells were categorized as live, and completely or partially red cells were classified as dead.

### 2.11 Evaluation of Sperm DNA Fragmentation

Sperm DNA fragmentation (SDF) was assessed as a measure of its integrity. In brief, 20 μL of each sample was mixed well with melted agarose and 10 μL of it was placed on a pre‐coated slide. A coverslip was then placed on each sample and allowed to solidify at 4°C for 10 min. The coverslips were then removed, and 2 drops of the denaturing solution were added to each slide. After 7 min, the samples were washed with water and treated with 2 drops of the lysing solution. After 10 min of incubation, the slides were washed with water and treated with 2 drops of 96% ethanol for 5 min. Following another wash with water, the samples were treated with a staining solution for 15 min. Finally, the samples were washed again with water, and 300 spermatozoa from each sample were evaluated with a light microscope (400×) (Olympus BX‐40; Olympus U‐RFL‐T, Tokyo, Japan). Sperm that had a pronounced halo were classified as sperm with intact DNA, while sperm with a small halo or no halo were classified as having fragmented DNA [27].

### 2.12 NO Measurement

The Griess test was used to quantify the concentration of NO [28]. To begin, the supernatant from the samples was obtained by centrifuging at 300 *g* for 10 min. Next, 100 μL of Griess reagent, which consists of 1% sulfanilamide and 0.1% N‐(1‐naphthyl)ethylenediamine dihydrochloride in 2% phosphoric acid, was added to 100 μL of the supernatant. The mixture was then incubated for 10 min at 37°C. Finally, the samples were read at 540 nm, and the concentrations of NO^2−^/NO^3−^, which are stable products of nitric oxide, were calculated using a sodium nitrite standard curve.

### 2.13 Measurement of the Activity of Antioxidant Enzymes

#### 2.13.1 GPx

The Glutathione Peroxidase Assay Kit (Navand Salamat Co., Urmia, Iran) was utilized to measure the activity of GPx. First, sperm samples (1 × 10^6^) were sonicated in ice‐cold PBS to obtain a cell lysate. The samples were then centrifuged at 9000 *g* for 15 min at 4°C. Subsequently, 50 μL of the supernatant was mixed with 40 μL of H_2_O_2_. The samples were incubated at room temperature for 15 min before adding 10 μL of NADPH to each sample. Finally, the initial absorbance readings were taken at 340 nm using a plate reader (Synergy H1, BioTek Co., USA). A second reading was performed after an additional 15 min, and the GPx activity was calculated using a standard curve for NADPH [29].

#### 2.13.2 CAT

CAT activity was measured using a commercial kit (Kushan Zist, Iran). First, sperm samples (1 × 10^6^) were sonicated in ice‐cold PBS to obtain a cell lysate. The samples were then centrifuged at 10000 *g* for 15 min, and the supernatant was collected and kept on ice. Next, 30 μL of methanol was added to each supernatant and mixed thoroughly, followed by the addition of 20 μL of H_2_O_2_. The samples were incubated for 20 min on a shaker in the dark. After this, the stopper reagent was added to each sample, and they were shaken for 10 min at room temperature. Finally, 10 μL of the oxidizer reagent was added to each sample, and the samples were shaken for an additional 5 min. The absorbance was measured at 540 nm using a microplate reader (Synergy H1, Bio Tek Co., USA). The formaldehyde standard curve was used to calculate CAT activity.

#### 2.13.3 SOD

SOD activity was measured using a commercial kit from Kushan Zist, Iran. In summary, 1 × 10^6^ sperm cells were sonicated in ice‐cold PBS to induce lysis. The samples were then centrifuged at 14000 *g* for 5 min, and the supernatant was collected and kept on ice. Afterward, 200 μL of a 5% water‐soluble tetrazolium solution was added to 20 μL of the supernatant from each sample. Then, 20 μL of xanthine oxidase was added to each sample. The samples were then shaken for 15 s and incubated at 37°C for 20 min. Following the incubation period, the absorbance was read at 450 nm using a plate reader (Synergy H1, Bio Tek Co., USA). The amount of enzyme needed to achieve 50% dismutation of the superoxide radical was defined as one unit of SOD.

### 2.14 Real‐time‐PCR

The complete RNA of each sperm sample was isolated using a total RNA extraction kit (Parstous, Iran). The extracted RNA was then quantified, and the cDNA was produced by reverse transcription using a commercial kit (Parstous, Iran). Afterward, the expression of genes including *Bcl2*, *Bax*, *Casp3*, *Gpx*, *Cat*, *Sod1*, and *Sod2* was quantified by SYBR green quantitative real‐time polymerase chain reaction (qRT‐PCR, ABI ViiA 7; Applied Biosystems, Foster City, CA). The forward and reverse primers are indicated in Table 1.

**TABLE 1:** Forward and reverse primer sequences used in the qRT‐PCR assays.

Gene	Forward primer	Reverse primer
*Cat*	5′‐GCAGATACCTGTGAACTGTC‐3′	5′‐GTAGAATGTCCGCACCTGAG‐3′
*Sod2*	5′‐CAGACCTGCCTTACGACTATGG‐3′	5′‐CTCGGTGGCGTTGAGATTGTT‐3′
*Sod1*	5′‐AACCAGTTGTGTTGTCAGGAC‐3′	5′‐CCACCATGTTTCTTAGAGTGAGG‐3′
*Gpx*	5′‐CCTCAAGTACGTCCGACCTG‐3′	5′‐CAATGTCGTTGCGGCACACC‐3′
*Bcl2*	5′‐TGCTGCTATCCTGCCAAG‐3′	5′‐GTCTGTGTTCTTCATCGTTACTTC‐3′
*Bax*	5′‐TGCAGAGGATGATTGCTGAC‐3′	5′‐GATCAGCTCGGGCACTTTAG‐3′
*Casp3*	5′‐TGGTGATGAAGGGGTCATTTATG‐3′	5′‐TTCGGCTTTCCAGTCAGACTC‐3′
*β‐Actin*	5′‐TGACCCAGATCATGTTTGAGACC‐3′	5′‐CTCGTAGATGGGCACAGTGTGGG‐3′

qRT‐PCR was performed with an initial denaturation step at 95°C for 10 min. This was followed by 40 cycles, each consisting of three steps: denaturation at 95°C for 15 s, annealing at 60°C for 30 s, and extension at 72°C for 30 s. Standard curves were created using progressive dilutions of cDNA. The qRT‐PCR reaction mixture contained 20 nM of each primer, 10 ng of cDNA, and SYBR Green qPCR Master Mix (Ampliqon, Denmark), with a total final volume of 20 μL. The qRT‐PCR data were then analyzed using the 2^−ΔΔCT^ method. Expression levels were normalized by amplifying a housekeeping *β‐actin* gene.

### 2.15 Statistical Analysis

The data were shown as mean ± standard deviation (SD). Statistical analysis was carried out by SPSS 23 software (SPSS, Chicago, IL, USA). All data were checked for normality using the Shapiro–Wilk test and for homogeneity of variances with Levene's test. One‐way ANOVA followed by Tukey's post hoc was applied to compare the means. All tests were performed in triplicate, and significance was considered at *p* < 0.05.

## 3 Results

### 3.1 Physicochemical Characteristics of SLNs

**FIGURE 1:**
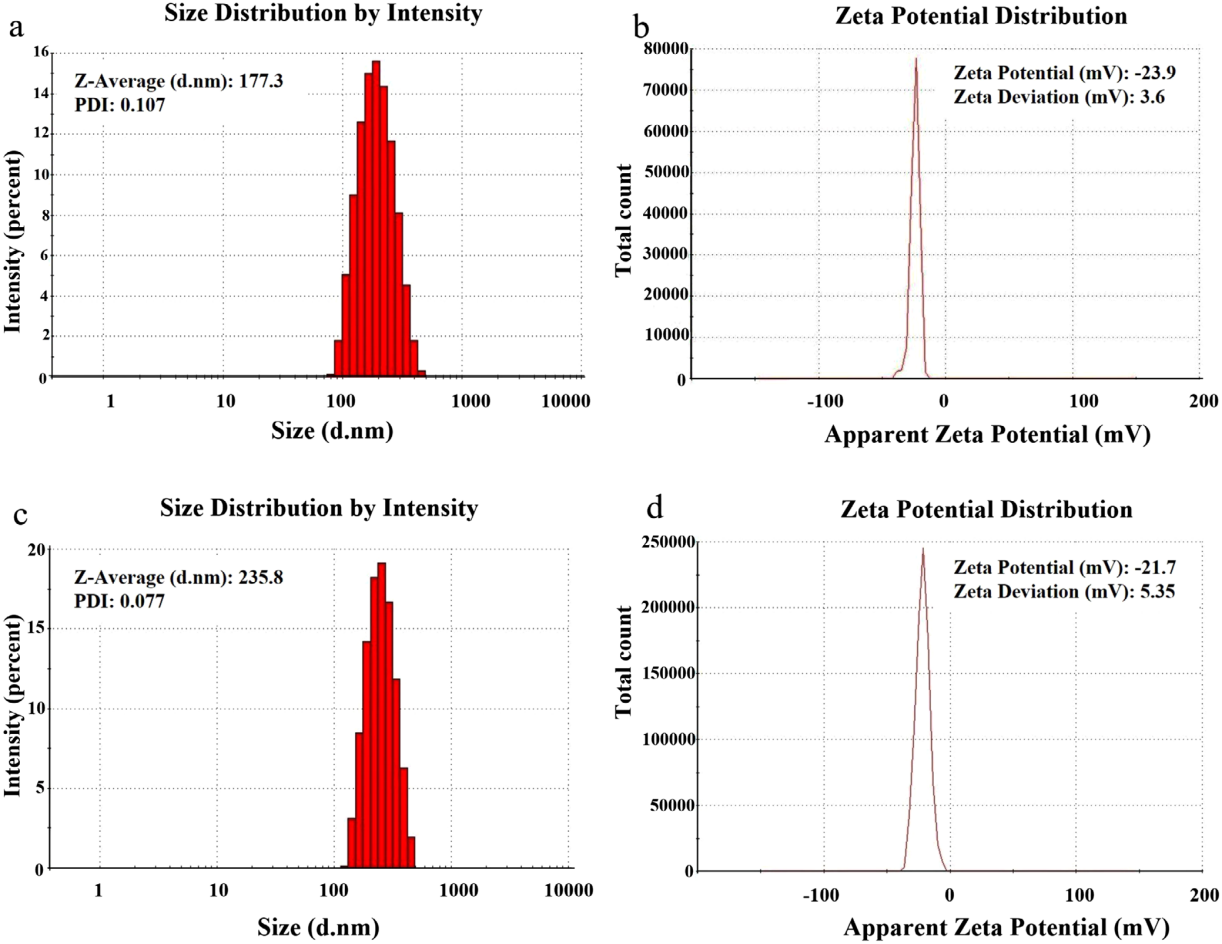
Size distribution of (a) B‐SLN with a size of 177.3 nm and (c) LHE‐SLN with a size of 235.8 nm. The zeta potential measurements demonstrated that (b) B‐SLN had a zeta potential of −23.9 mV, while (d) LHE‐SLN exhibited a zeta potential of −21.7 mV.

Table 2 displays the property data for the size, zeta potential, PDI, DL (%) and EE (%) of the SLNs. The size of B‐SLN was 177.3 ± 12.33 nm with a PDI of 0.107 ± 0.09. The size and PDI of LHE‐SLN were 235 ± 11.06 nm and 0.077 ± 0.01, respectively. The zeta potential was negative for both B‐SLN (−23.9 ± 3.6 mV) and LHE‐SLN (−21.7 ± 5.35 mV) (Figure 1). The EE (%) of LHE‐SLN was 59 ± 2 with a DL (%) of 13.49 ± 0.79.

**TABLE 2:** Characterization of the SLNs. Data is presented as the mean ± SD of three separate experiments.

	Size (nm)	PDI	Zeta potential (mV)	DL (%)	EE (%)
B‐SLN	177.3 ± 12.33	0.107 ± 0.09	−23.9 ± 3.6	—	—
LHE‐SLN	235.8 ± 11.06	0.077 ± 0.01	−21.7 ± 5.35	13.49 ± 0.79	59 ± 2.0

Abbreviations: DL, drug loading; EE, entrapment efficiency; PDI, polydispersity index.

### 3.2 SEM

**FIGURE 2:**
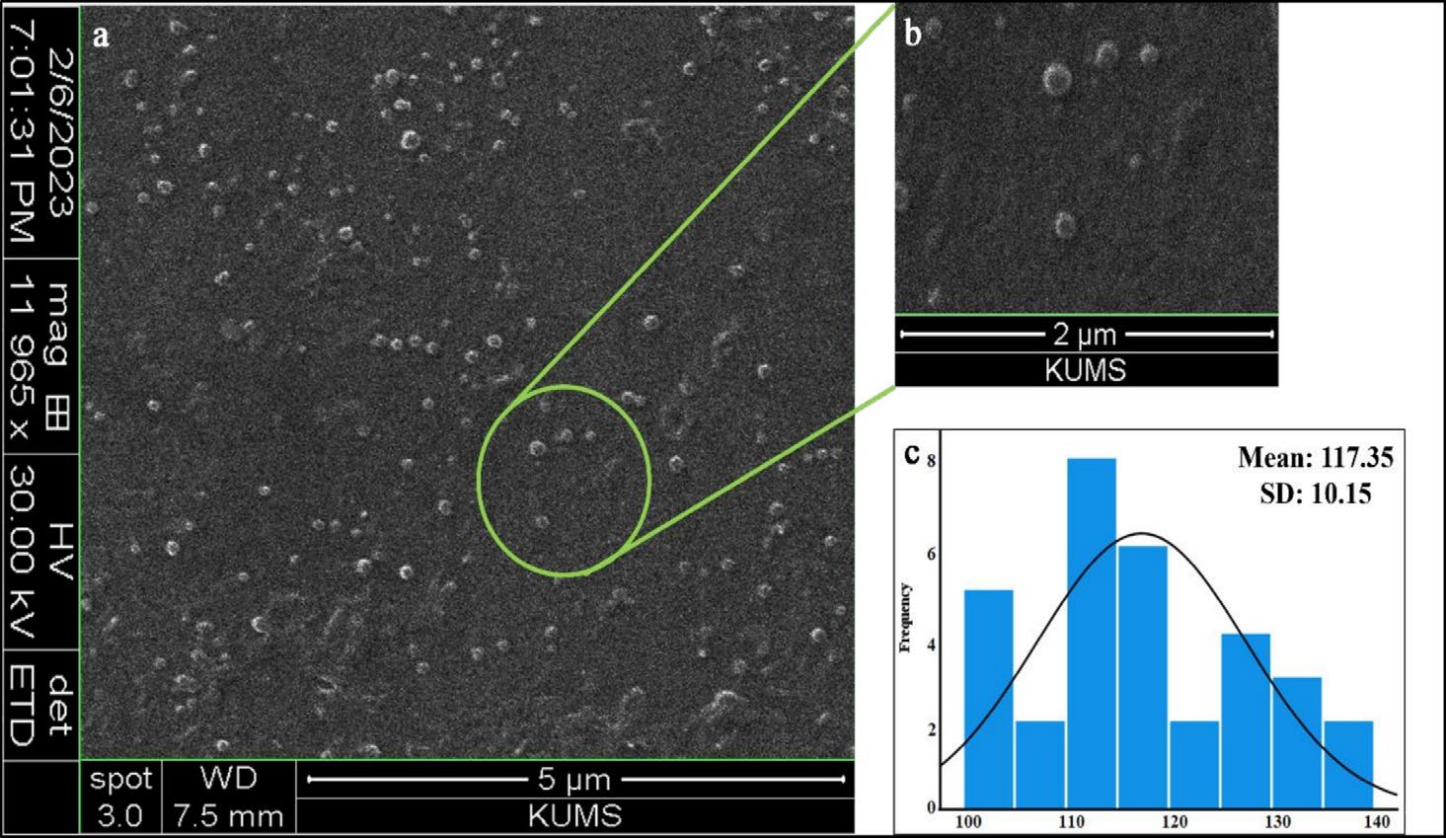
SEM images at different scales: (a) 5 μm and (b) 2 μm, suggesting that the morphology of the LHE‐SLN is completely spherical and uniformly distributed. The ImageJ analysis indicated an average size of 117.35 ± 10.15 nm (c).

The SEM image (Figure 2) showed spherical LHE‐SLN particles. This image also suggested that the particles were monodispersed with no aggregation, which aligns with the DLS results, indicating a low PDI. Analysis using ImageJ, where 32 randomly selected particles were counted, revealed an average particle size of about 117 nm.

### 3.3 FT‐IR

**FIGURE 3:**
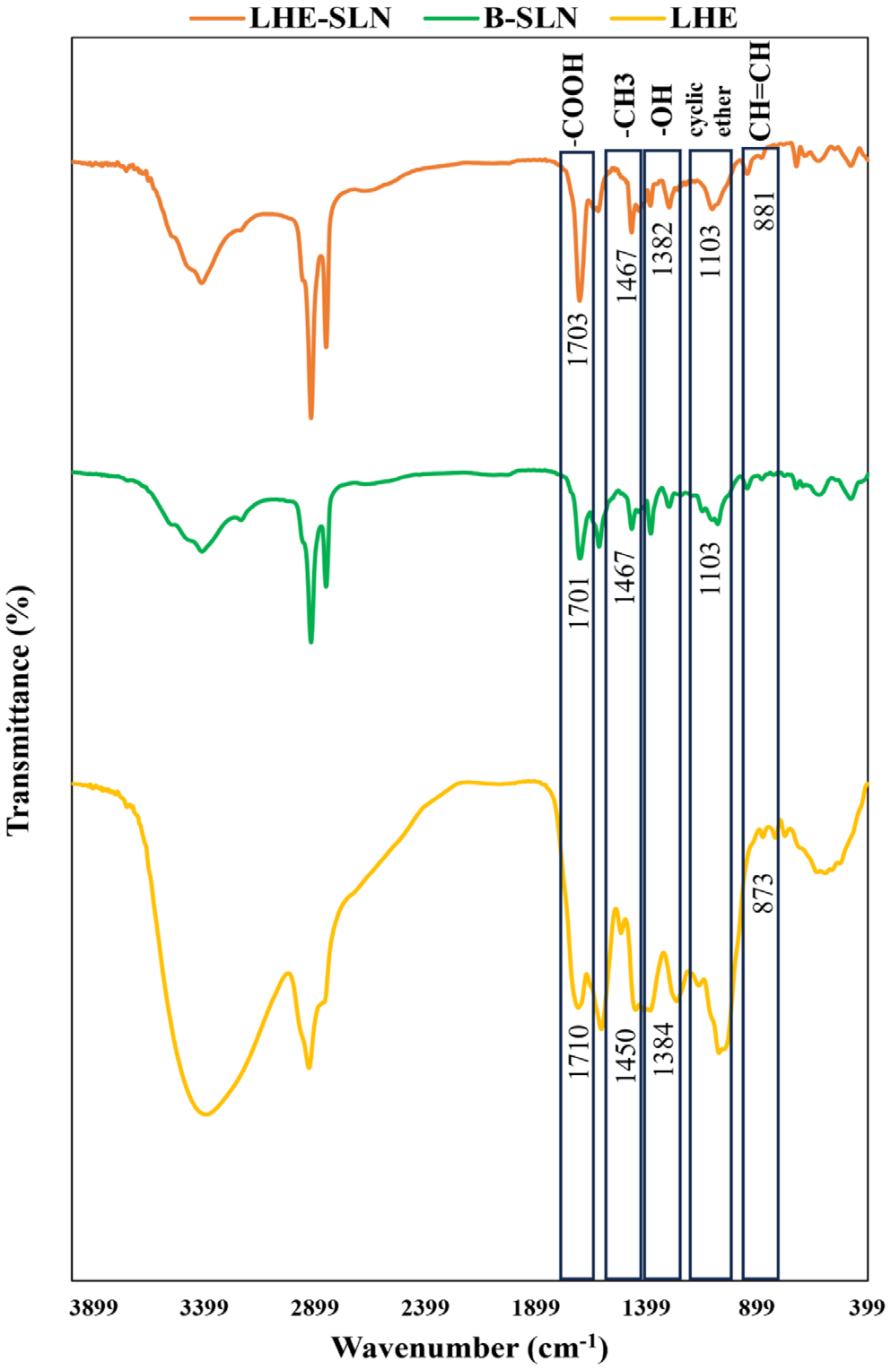
FT‐IR spectra of B‐SLN, pure LHE and LHE‐SLN. The results confirm the synthesis of LHE‐SLN.

Figure 3 indicates the FT‐IR spectra of the B‐SLN, pure LHE, and LHE‐SLN. The B‐SLN was synthesized using Tween 80 and stearic acid; hence, the obtained peaks belonged to these molecules. Absorption at 1701, 1614, 1103, 1078, and 723 refers to carboxylic acid, alkenyl C=C stretch, cyclic ether, C—O stretch, and methylene, respectively, and has been found for B‐SLN.

In the spectrum of pure LHE, sharp bands were detected at 2927, 1710, 1450, 1384, 1051, and 873, which belong to C—H stretching, carboxylic acid, methyl, OH bend, C—O stretching, and unsaturated vinyl (CH=CH), respectively.

LHE‐SLN had absorption at 1703 that corresponded to carboxylic acid. In addition, the peaks at 1620, 1467, 1382, 1103, 881, and 723 refer to alkenyl C=C stretch, methyl, OH bend, cyclic ether, unsaturated vinyl (CH=CH), and methylene, respectively. The presence of common peaks confirmed the synthesis of LHE‐SLN.

### 3.4 In Vitro Drug Release and Release Kinetic Study

**FIGURE 4:**
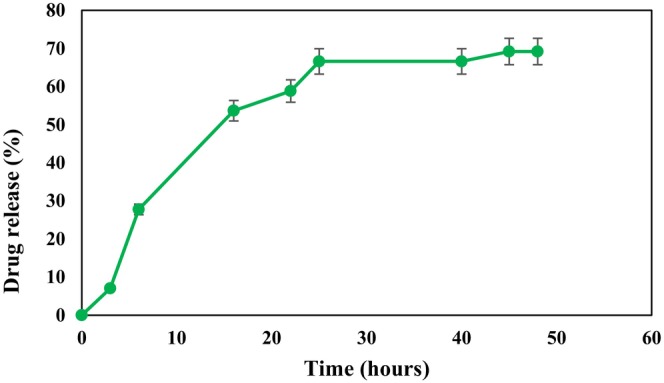
In vitro drug release of LHE‐SLN in PBS: pH 7.4, over 48 h.

Figure 4 represents the pattern of LHE release from SLN over time. The experiment indicated that on the first day, there was a gradual release with a gentle slope, reaching 66% after 25 h. This was followed by a period of constant release over the next 23 h. The maximum release of the extract, 69.19%, was observed in the last hour of the experiment.

**FIGURE 5:**
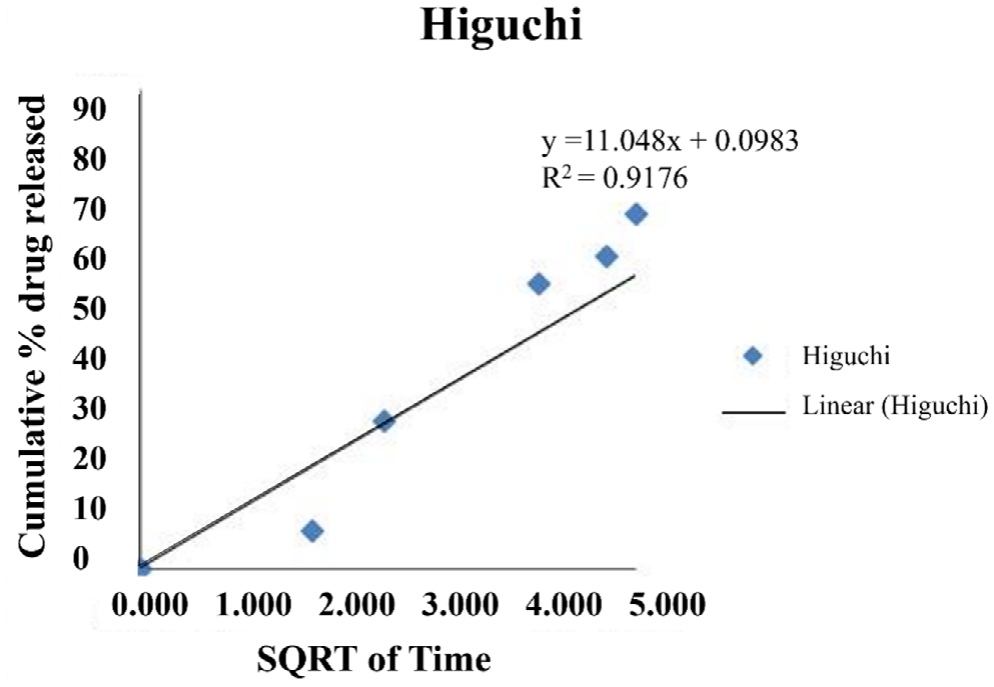
The Higuchi model indicates drug release kinetics of LHE‐SLN.

The LHE release kinetics from SLN were examined to determine the mechanism of drug release. The *R* ^2^ values for each model are shown in Table 3. Generally, the model with the highest *R* ^2^ value is considered the most suitable for representing the drug release mechanism. As shown in Figure 5, the release kinetics of LHE‐SLN were best fitted to the Higuchi model, as there was greater linearity (*R* ^2^ = 0.9176) for the Higuchi model of release kinetics compared to other models, including zero‐order, first‐order, and Korsmeyer‐Peppas.

**TABLE 3:** Kinetics of in vitro drug release. The release kinetics of LHE‐SLN were best fitted to the Higuchi model.

R^2^					
	Zero order	First order	Higuchi	Hixson‐Crowell	Korsmeyer–Peppas
LHE‐SLN	0.7783	0.8514	0.9176	0.8282	0.8949

### 3.5 Assessments of Sperm Motility, Viability, and SDF (%)

**FIGURE 6:**
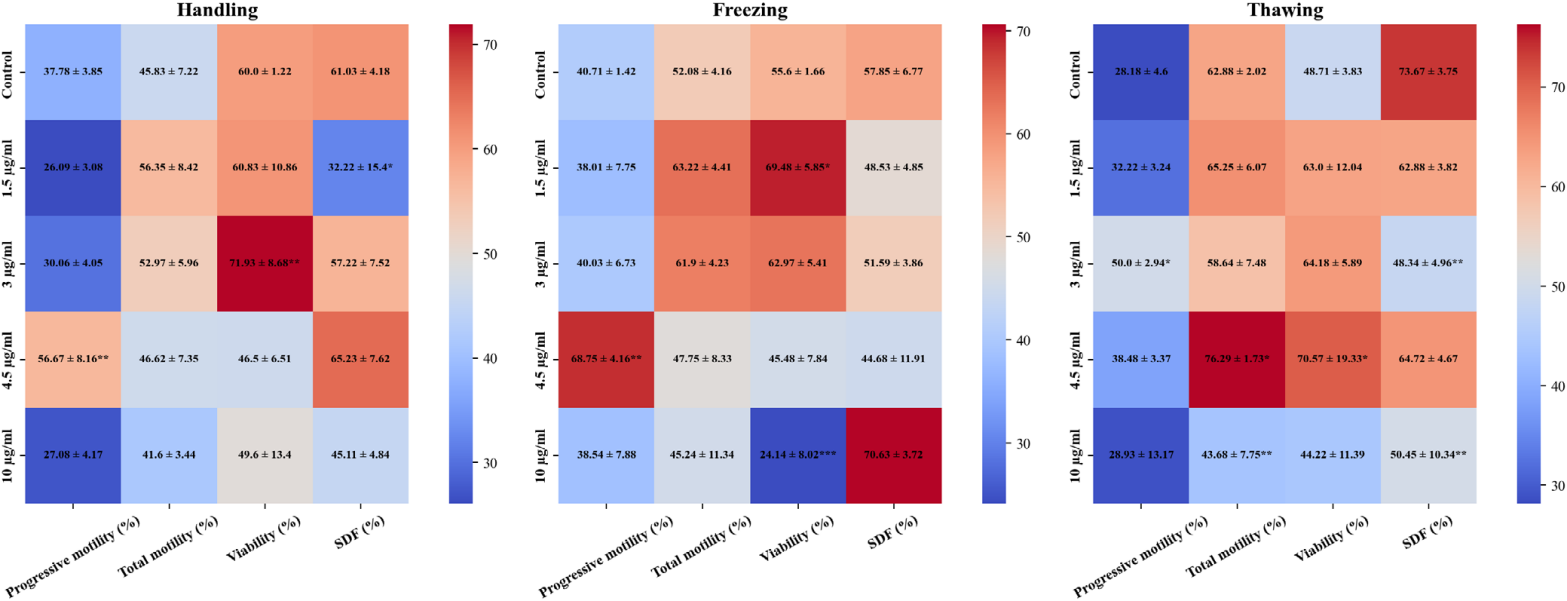
Heatmap illustrating progressive motility, total motility, viability, and sperm DNA fragmentation (SDF) of NMRI mouse sperm during handling, freezing, and thawing after treatment of the respective medium with LHE‐SLN. Each cell represents mean ± SD. Color intensity corresponds to the raw mean values (blue = low, red = high), without data normalization or standardization. The color scale was kept consistent across all parameters for direct comparison. Asterisks denote statistical significance vs. the control group (*p < 0.05, **p < 0.01, ***p < 0.001).

Figure 6 presents the progressive and total motility of spermatozoa after exposure to various concentrations of LHE‐SLN during the handling, freezing, and thawing processes. The results indicated that the addition of 4.5 μg/mL of LHE‐SLN to the handling and freezing media enhanced the progressive motility of sperm. Additionally, adding 3 μg/mL to the thawing media also improved progressive motility compared to the control group. There was an increase in the percentage of sperm exhibiting total motility after treatment with 1.5 and 3 μg/mL of LHE‐SLN in the handling and freezing media, although this increase was not statistically significant. However, after thawing the mouse spermatozoa in the presence of LHE‐SLN, an improvement in total motility was observed at a concentration of 4.5 μg/mL, while a decrease was noted at a concentration of 10 μg/mL.

The results of the viability assessment showed that in the handling phase, the 3 μg/mL concentration of LHE‐SLN significantly enhanced sperm viability compared to the control group, with a notable increase from 60.83% ± 10.86% to 71.93% ± 8.68% (*p* < 0.01). During the freezing phase, sperm viability improved with the 1.5 μg/mL LHE‐SLN and decreased after exposure to 10 μg/mL, compared to the control group. Post‐thaw viability was significantly higher in the 4.5 μg/mL LHE‐SLN group compared to the control group (Figure 6).

The measurement of SDF (%) revealed that during the handling phase, treatment with 1.5 μg/mL LHE‐SLN significantly reduced SDF (%). In the freezing phase, there was no significant change in SDF (%) after treatment with LHE‐SLN. However, after thawing, the SDF (%) was significantly lower in the groups treated with 3 μg/mL and 10 μg/mL LHE‐SLN compared to the control group (Figure 6).

### 3.6 NO Production

**FIGURE 7:**
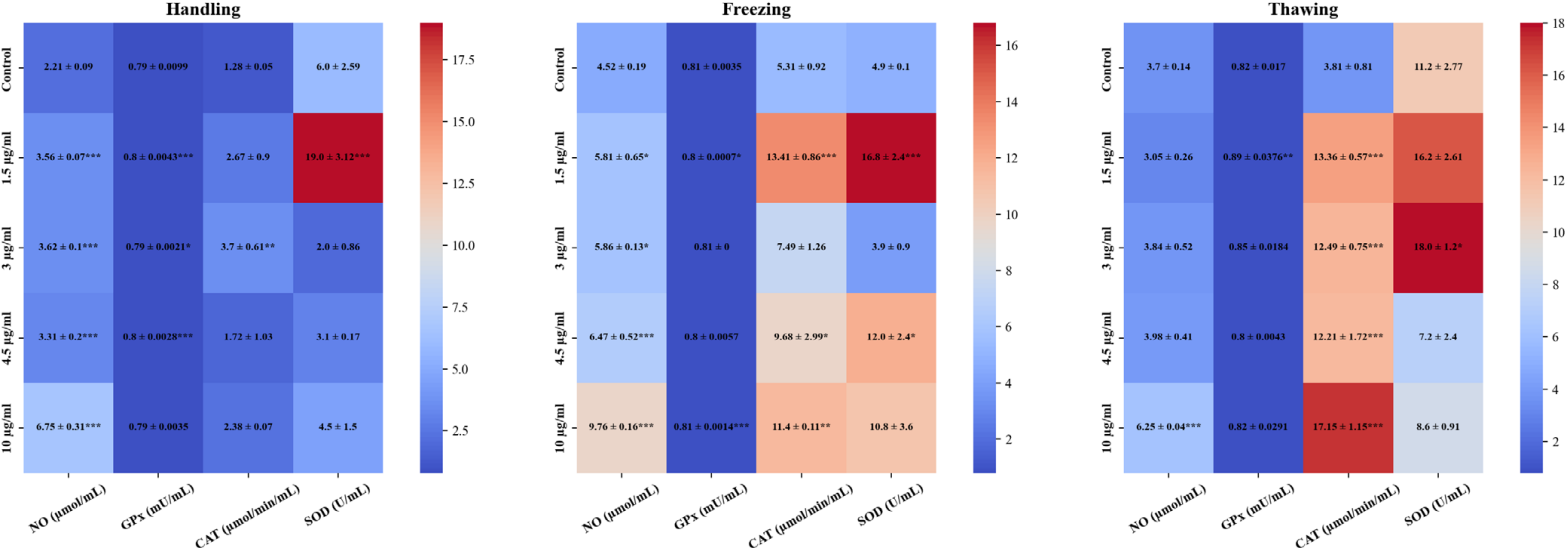
Heatmap illustrating the effects of LHE‐SLN on antioxidant enzyme activities (CAT, SOD, GPx) and nitric oxide (NO) levels in NMRI mouse sperm during handling, freezing, and thawing. Each cell represents mean ± SD. Color intensity corresponds to the raw mean values (blue = low, red = high), without data normalization or standardization. The color scale was kept consistent across all parameters for direct comparison. Asterisks denote statistical significance vs. the control group (*p < 0.05, **p < 0.01, ***p < 0.001).

Figure 7 displays the results of the Griess test conducted after adding LHE‐SLN to the media used for sperm handling, freezing, and thawing. The findings showed that all concentrations of LHE‐SLN led to a significant increase in NO production in both the handling and freezing media of sperm. Only the use of the highest dose resulted in a significant elevation in NO levels during the thawing stage compared to the control (6.25 ± 0.04 vs. 3.70 ± 0.14 μmol/mL; *p* < 0.001).

### 3.7 GPx Activity

Figure 7 presents the activity of GPx in mouse sperm following treatment with various concentrations of LHE‐SLN in the handling, thawing, and freezing media. Enzyme activity significantly increased in the handling media at concentrations of 1.5, 3, and 4.5 μg/mL of LHE‐SLN. During the freezing stage, only the lowest dose caused a slight reduction in GPx activity (*p* < 0.05), while a concentration of 10 μg/mL significantly increased it (0.8142 ± 0.0014; *p* < 0.001). Moreover, the addition of LHE‐SLN during the thawing process resulted in enhanced GPx activity at a concentration of 1.5 μg/mL, with minimal variation observed at other concentrations.

### 3.8 CAT Activity

The activity of catalase (CAT) was assessed after treating sperm with various concentrations of LHE‐SLN during handling, freezing, and thawing. The results, summarized in Figure 7, show that a concentration of 3 μg/mL of LHE‐SLN enhanced CAT activity in the handling media. Additionally, when mouse sperm was exposed to 1.5, 4.5, and 10 μg/mL of LHE‐SLN during cryopreservation, there was a notable increase in CAT activity. After thawing, CAT activity was significantly higher at all doses compared to the control, with the highest level observed at 10 μg/mL (17.15 ± 1.15 μmol/min/mL; *p* < 0.001).

### 3.9 SOD Activity

The results of measuring SOD activity after treating the handling, freezing, and thawing media of mouse sperm with LHE‐SLN are presented in Figure 7. The activity of this enzyme increased following the addition of 1.5 μg/mL of LHE‐SLN to the handling media. Similar increases were observed with 1.5 μg/mL and 4.5 μg/mL of LHE‐SLN in the freezing media. However, other doses had minor or non‐significant effects, except for a modest rise at 3 μg/mL during thawing (18 ± 1.2 U/mL; *p* < 0.05), suggesting a non‐linear dose–response pattern.

### 3.10 The Impact of LHE‐SLN Treatment on the Expression of Apoptotic and Antioxidant‐Related Genes in the Sperm of NMRI Mice

**FIGURE 8:**
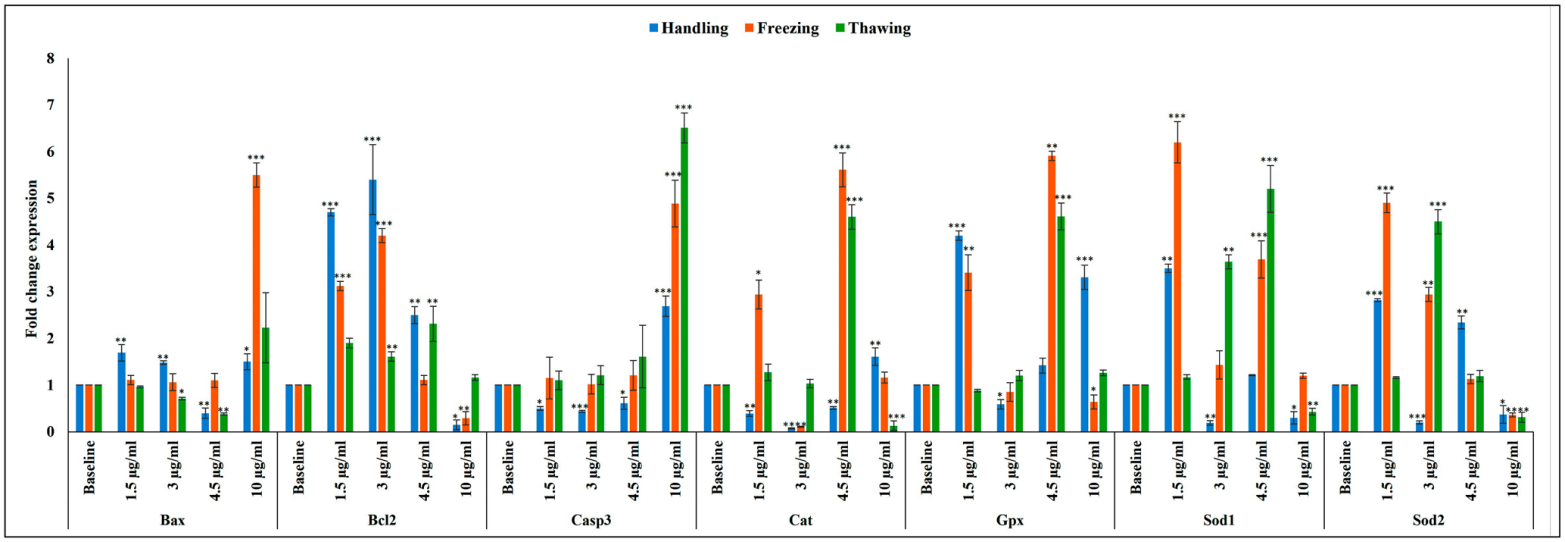
Relative fold change determined by quantitative real‐time PCR (qRT‐PCR) analysis of antioxidant enzymes (Sod1, Sod2, Cat, Gpx), and apoptosis‐related genes (Bax, Casp3, and Bcl2) after treatment of handling, freezing, and thawing media of NMRI mouse sperm with different concentrations of LHE‐SLN. All data were normalized with β‐Actin expression and represented as relative to baseline; data were represented as mean ± SD. *p ≤ 0.05; **p ≤ 0.01, ***p ≤ 0.001.

After treating the media with LHE‐SLN, the expression of *Bax* initially increased significantly at concentrations of 1.5 and 3 μg/mL but decreased at 4.5 μg/mL. In contrast, *Bcl2* expression was significantly elevated at 1.5, 3, and 4.5 μg/mL, but it dramatically declined at 10 μg/mL. *Casp3* expression significantly increased at 10 μg/mL, while it decreased at 1.5, 3, and 4.5 μg/mL of LHE‐SLN. *Cat* expression was lowest at 3 μg/mL and highest at 10 μg/mL. *Gpx* peaked at both 1.5 and 10 μg/mL. Additionally, *Sod1* and exhibited similar biphasic patterns, with peak values at 1.5 μg/mL, followed by significant declines at 3 and 10 μg/mL (Figure 8).

As shown in Figure 8, after treating NMRI mouse sperm with LHE‐SLN in the freezing process, there were no significant changes in *Bax* expression until a sharp increase was observed at a concentration of 10 μg/mL. *Bcl2* levels remained elevated at concentrations of 1.5 and 3 μg/mL but declined significantly at 10 μg/mL. *Casp3* expression was highest at 10 μg/mL. Antioxidant genes showed significant upregulation at 1.5 and 4.5 μg/mL for *Cat*, *Gpx*, and *Sod1*, and at 1.5 and 3 μg/mL for *Sod2*. However, a concentration of 10 μg/mL appeared to reduce the expression of *Gpx* and *Sod2*.

During the thawing step, *Bax* expression decreased at concentrations of 3 and 4.5 μg/mL, whereas *Bcl‐2* expression increased after treatment of the thawing media with these doses. The expression of *Cat* and *Gpx* reached its maximum upregulation at 4.5 μg/mL. Notably, *Casp3* expression increased significantly at a concentration of 10 μg/mL. *Sod1* expression peaked at both 3 and 4.5 μg/mL, while *Sod2* expression was elevated at 3 μg/mL. However, the expression levels of *Sod1*, *Sod2*, and *Cat* significantly decreased at 10 μg/mL (Figure 8).

## 4 Discussion

This study evaluated the cryoprotectant and antioxidant capacity of LHE‐SLN to assess its ability to supplement NMRI mouse sperm during the preservation, freezing, and thawing processes.

The size assessment of LHE‐SLNs yielded promising results, with a size of less than 250 nm and an exceptionally low PDI of approximately 0.077. Previous research indicates that nanoparticles within the range of 50 to 300 nm are efficiently absorbed by cells through active endocytosis mechanisms [30]. A monodispersed population of nanocarriers within this size range contributes to their stability and effectiveness. In this context, lipid‐based nanocarriers demonstrate optimal drug delivery efficacy when their PDI is below 0.3 [31]. The small and homogeneous size of our carrier suggests strong potential as a robust drug delivery system.

Our nanoparticles exhibited a spherical shape as observed in the SEM images, and they also had a negative zeta potential. This finding aligns with previous studies that indicate SLNs are small spherical particles with a solid lipid core at room temperature. Additionally, the stability of SLNs in suspension is directly influenced by their zeta potential. A highly negative or positive surface charge leads to electrostatic repulsion between similarly charged particles, which enhances their stability and reduces aggregation. Generally, negative surface charges contribute to greater stability compared to positive charges [32, 33].

The in vitro release profile of LHE showed an initial sustained release followed by a steady phase. Based on this profile, we hypothesized that the initial release of LHE was due to the drug molecules binding to the surface of the carrier, while the subsequent phase involved the release of encapsulated drug from the lipid core of the carrier. Mathematical modeling of the release kinetics indicated that the Higuchi model provided the best fit (*R* ^2^ = 0.9176). This model suggests that the controlled matrix diffusion of LHE is the predominant mechanism of drug release, which aligns with the characteristics of matrix‐based systems and indicates a non‐Fickian diffusion mechanism [34]. This analysis revealed that the rate‐limiting step for LHE release is the diffusion through the lipid matrix, which maintains sustained drug levels over time. The Higuchi model has also been validated in studies involving plant extract‐loaded SLN [35].

We also investigated how LHE‐SLN affects sperm motility during handling, cryopreservation, and thawing. In general, incorporating LHE‐SLN into the handling, freezing, and thawing media enhanced sperm progressive motility. Furthermore, the inclusion of LHE‐SLN in the freezing and thawing media improved the total motility of sperm. Research has shown that OS leads to axonemal and morphological defects in sperm, causes lipid peroxidation of the sperm membrane, and results in mitochondrial dysfunction, all of which contribute to reduced sperm motility [36]. It is indicated that the hydroalcoholic extract of lavender possesses antioxidant properties, such as the ability to scavenge free radicals. This is because the extraction process and the solvents used can effectively extract polyphenols, which are the main antioxidant compounds found in lavender [37]. Therefore, the antioxidant properties of this extract help combat OS and improve sperm motility.

Furthermore, LHE‐SLN enhanced sperm viability, particularly at 3 μg/mL for handling, and at 1.5 and 4.5 μg/mL during freezing and thawing processes, respectively. Apoptosis plays a crucial role in reducing sperm viability following OS, particularly during cryopreservation [38]. According to studies, LHE contains a variety of polyphenols, including flavonoids, phenolic acids, stilbenes, and lignans, which possess antioxidant properties due to their ability to scavenge free radicals, inhibit the activity of pro‐oxidative enzymes, and chelate metal ions [13]. Farhadi et al. found that adding LHE to semen extenders significantly reduced MDA and H_2_O_2_ levels in cryopreserved ram epididymal sperm [39]. Previous research has suggested that LHE reduces cell apoptosis, Bax/Bcl‐2 ratio, and ROS levels in neural cells [40]. It has also been found that the hydroalcoholic extract of lavender decreases Caspase‐3 levels and apoptosis in mice with epilepsy [41]. In our study, LHE was found to protect normal spermatozoa from apoptosis in an environment affected by OS.

The SDF (%) was reduced after treating the handling and thawing media of sperm with LHE‐SLN. Specifically, a concentration of 1.5 μg/mL during handling and 3 and 10 μg/mL during thawing showed significant results. One previous study by Farhadi et al. reported that 1.5 and 3 μg/mL of lavender ethanolic extract decreased the SDF percentage of post‐thawed ram epididymal sperm [39]. While they observed these results after supplementing frozen sperm with lavender ethanolic extract, we did not see any significant changes in this process, possibly due to the species we used (mice).

Although LHE‐SLN seemed to have an impact on sperm motility, viability, and SDF (%), there was considerable variability in the results, and some effects did not reach statistical significance. This variability may be attributed to the limited sample size in the current study, which could explain the lack of a consistent dose–response relationship, limiting the statistical strength of the conclusions. It is important to acknowledge this limitation and interpret the findings with caution. Future studies with larger sample sizes are needed to confirm these preliminary results and draw more definitive conclusions.

LHE‐SLN enhanced NO production in the handling and freezing media at all tested concentrations. NO exerts a dual role in sperm function. At low levels, it improves sperm motility and facilitates the acrosome reaction by acting as a radical scavenger. In contrast, at moderate to high concentrations, NO can impair motility by reacting with superoxide anions to form peroxynitrite, a highly reactive molecule that disrupts sperm function [42, 43]. This negative effect has been observed at a concentration of 10 μg/mL during handling processes.

The activity of antioxidant enzymes in mouse sperm, including CAT, SOD, and GPx, significantly improved following treatment with LHE‐SLN in the respective media during the handling, freezing, and thawing processes. The expression levels of *Cat*, *Sod1*, *Sod2*, and *Gpx* were notably increased, too. Farhadi et al. found that adding lavender ethanolic extract to semen extenders could enhance the activity of SOD and GPx in cryopreserved ram epididymal sperm [39]. The primary aroma constituents of the 70% ethanolic extracts of lavender are linalool and linalyl acetate, which provide this extract with strong antioxidant properties [44]. Bar et al. reported that linalool increased CAT activity in diabetic rats experiencing OS, further supporting the antioxidant potential of lavender extract [45]. Altinoz et al. discovered that linalool could reduce doxorubicin‐induced oxidative stress in rats by elevating the activity of CAT [46]. Additionally, treated fibroblasts with linalool showed a significant reduction in the depletion of GPx after UVB irradiation [47]. Furthermore, the SLN delivery system likely enhances the bioavailability and stability of the components in lavender ethanolic extract, as nanoencapsulation has been found to increase the therapeutic efficacy of bioactive compounds [48]. Our nanocarriers allow sperm to be exposed to a consistent amount of LHE, especially throughout the cryopreservation process.

Treatment of mouse sperm with LHE‐SLN could significantly reduce the expression of *Bax* and *Casp3* as proapoptotic genes and increase the *Bcl2* expression as an antiapoptotic gene. Mohamed et al. indicated that linalool reduced the expression of apoptotic markers such as *Bax*, *Casp9*, and *Casp3*, while increasing the expression of the anti‐apoptotic gene *Bcl2* in male Wistar rats [49]. Aghaz et al. showed that SLNs have significant potential for delivering resveratrol and melatonin to embryos, which influence their gene expression. Specifically, the drug loaded in SLNs was able to downregulate the pro‐apoptotic genes *Bax* and *Casp3*, while also increasing the expression of the *Bcl2* gene [50]. However, we discovered that at high concentrations, the outcome is reversed. This finding aligns with previous studies, such as those by Tayarani‐Najaran et al., who reported that the ethanolic extract of lavender upregulated *Bax* expression in HeLa cells compared to a control group [51]. Moreover, Mojodi et al. found that the expression of the *Casp3* gene increased approximately two‐fold in the MCF‐7 and SK‐BR‐3 cell lines after treatment with lavender essential oil‐synthesized nanoparticles at high doses, while *Bcl2* expression decreased [52]. In addition, Abdelnour et al. demonstrated that propolis‐loaded nanoliposomes (RPNL), as an antioxidant, at 2 μg/mL reduced *Bax* and *Casp3* expression in buffalo sperm during the freezing process, while higher concentrations (4–6 μg/mL) showed a non‐significant increase in expression compared to the 2 μg/mL dose [53]. Furthermore, excessive amounts of antioxidants have been associated with an increased risk of chromosomal abnormalities through interference with endogenous DNA repair pathways and the upregulation of pro‐apoptotic genes [54, 55]. Although the absence of a B‐SLN control group is one of the limitations of this study, previous studies on NMRI mouse sperm during storage, freezing, and thawing have shown that B‐SLN exhibited no toxicity, even at high concentrations [21]. Therefore, the detrimental effects of LHE‐SLN at 10 μg/mL are more likely attributable to the elevated levels of phenolic compounds present in LHE. At high concentrations, these polyphenols may act as pro‐oxidants, disrupting cellular homeostasis by damaging proteins, lipids, and DNA, ultimately leading to cell death [56]. In addition to these potential mechanisms, the specific molecular pathways responsible for the toxicity observed at high doses of LHE‐SLN were not directly explored in this study. We recognize this as a limitation of our research and suggest that future investigations should focus on elucidating these pathways to gain a more comprehensive understanding.

## 5 Conclusion

The study utilized SLN to deliver LHE to NMRI mouse sperm during handling, cryopreservation, and thawing processes. The SLN‐LHE exhibited promising physicochemical properties, including a small size, low PDI, spherical shape, and a controlled drug release profile. Exposure to SLN‐LHE during these procedures improved sperm parameters such as motility, viability, and DNA integrity. Additionally, the production of NO and the activity of antioxidant enzymes, CAT, GPx, and SOD, increased following these interventions. These findings should be considered preliminary, as the effects were not always statistically significant or dose‐dependent across all parameters. Further investigation with larger sample sizes and improved experimental designs is necessary to validate and expand upon these observations. Furthermore, there was an increase in the gene expression of antioxidant enzymes (*Cat, Sod1, Sod2, and Gpx*), while the expression of pro‐apoptotic genes *Bax* and *Casp3* decreased, along with an elevation in the expression of the anti‐apoptotic gene *Bcl2*. Treatment with a high dose (10 μg/mL) increased pro‐apoptotic gene expression, while some sperm parameters improved, highlighting the need for further studies to assess the potential toxicity of high doses. Overall, the results indicated the notable antioxidant capacity of LHE and the potential of SLN as an effective nanosystem for transporting and delivering these compounds to NMRI mouse sperm.

## Ethics Statement

This study was approved by the Research Ethics Committee (Ethics Code: IR.NIMAD.REC.1402.045).

## Conflicts of Interest

The authors declare no conflicts of interest.

## Data Availability

The data that support the findings of this study are available on request from the corresponding author. The data are not publicly available due to privacy or ethical restrictions.
